# High Sensitivity Flexible Electronic Skin Based on Graphene Film

**DOI:** 10.3390/s19040794

**Published:** 2019-02-15

**Authors:** Xiaozhou Lü, Jiayi Yang, Liang Qi, Weimin Bao, Liang Zhao, Renjie Chen

**Affiliations:** 1 The School of Aerospace Science and Technology, Xidian University, Xi’an 710071, China; lxz@uw.edu (X.L.); lqi_1@stu.xidian.edu.cn (L.Q.); baoweimin@cashq.ac.cn (W.B.); 2 Science and Technology on Space Physics Laboratory, Beijing 100076, China; imu-zhl@163.com; 3 Beijing Key Laboratory of Environmental Science and Engineering, School of Chemical Engineering and the Environment, Beijing Institute of Technology, Beijing 100081, China; chenrj@bit.edu.cn

**Keywords:** aerospace, flexible sensor, micro-pressure measurement, sensitivity compensation

## Abstract

Electronic skin with high sensitivity, rapid response, and long-term stability has great value in robotics, biomedicine, and in other fields. However, electronic skin still has challenges in terms of sensitivity and response time. In order to solve this problem, flexible electronic skin with high sensitivity and the fast response was proposed, based on piezoresistive graphene films. The electronic skin was a pressure sensor array, composed of a 4×4 tactile sensing unit. Each sensing unit contained three layers: The underlying substrate (polyimide substrate), the middle layer (graphene/polyethylene terephthalate film), and the upper substrate bump (polydimethylsiloxane). The results of the measurement and analysis experiments, designed in this paper, indicated that the flexible electronic skin achieved a positive resistance characteristic in the range of 0 kPa–600 kPa, a sensitivity of 10.80Ω/kPa in the range of 0 kPa–4 kPa, a loading response time of 10 ms, and a spatial resolution of 5 mm. In addition, the electronic skin realized shape detection on a regular-shaped object, based on the change in the resistance value of each unit. The high sensitivity flexible electronic skin designed in this paper has important application prospects in medical diagnosis, artificial intelligence, and other fields.

## 1. Introduction

Electronic skin is an electronic system which can simulate the perception of human skin [1,2,3]. As electronic skin has the ability to detect pressure [4,5,6,7,8,9], it has received widespread attention in the fields of human health monitoring [10,11,12], medical diagnosis [13], biological research [14,15], artificial intelligence, and other fields [10,16]. In order to simulate the human skin pressure sensing system, electronic skin should be able to measure different pressure ranges (less than 10 kPa, and 10–100 kPa), and has the characteristics of high sensitivity and a fast response time [10]. Various approaches have been used to develop electronic skin. The most common working principles of electronic skin are piezocapacitance and piezoresistance. Capacitive electronic skin usually has high sensitivity. Joo et al. presented a pressure sensitive transistor array, based on a carbon nanotube (CNT) silver nanowire. The array had a sensitivity of 9.9 kPa${}^{-1}$ in the range of 0–0.6 kPa and could be easily integrated into a wearable system [17]. Lipomi et al. designed an electronic skin which can measure pressure and tension. This electronic skin could bear up to 150% of the strain, and had conductivity under the tensile state as high as 2200 S/cm, in the range of 0–50 kPa with a sensitivity of 0.004 kPa${}^{-1}$ [18]. Although the capacitive electronic skin had high sensitivity, it was vulnerable to stray capacitance and parasitic capacitance, the measurement of the electronic skin was unstable, and the measuring range was small. Electronic skin based on resistance has the advantages of high sensitivity, good chemical stability, and a simple manufacturing process. Yu et al. proposed a high-performance piezoresistive electronic skin using CNT and polydimethylsiloxane (PDMS) [19]. The electronic skin could measure pressure in the range of 7–50 kPa, and had a response speed of 10 ms and good stability. However, the sensitivity of the electronic skin was only −0.101±0.005 kPa${}^{-1}$ (<1 kPa). Gong et al. presented an gold nanowires (AuNWs) -based pressure sensor, which had a sensitivity of 1.14 kPa${}^{-1}$ in the range of 13 Pa–50 kPa [20].

Based on the above analysis, although the stability of the piezoresistive electronic skin is good, the sensitivity is relatively low. Therefore, to fabricate an electronic skin with high sensitivity, wide measuring range, fast response time, good stability, and a high spatial resolution still provides many challenges.

In order to solve the above problems, a flexible electronic skin, based on 2D graphene film, is proposed. The electronic skin is composed of 4×4 tactile sensing units which contain three layers: The lower substrate (polyimide substrate, or PI substrate), the piezoresistive layer (graphene/polyethylene terephthalate film), and the PDMS bump layer. This electronic skin has the advantages of high sensitivity, large measurement range, and fast response time, and has high practical values for the progress and development of artificial intelligence, rehabilitation medicine, and other fields.

## 2. Materials and Methods

### 2.1. Principle

**Figure 1:**
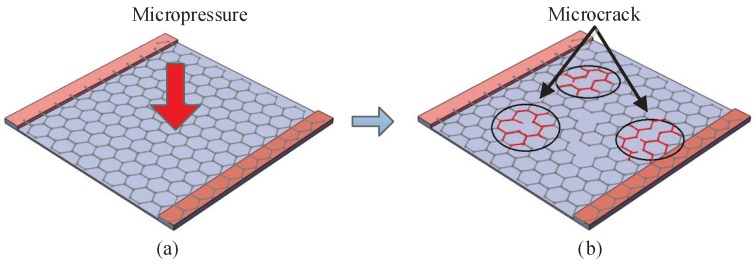
Schematic diagram of the working principle of the sensing unit. (**a**) Morphology of graphene thin films before being subjected to micropressure; (**b**) Microstructure of graphene film under compression.

The principle of the flexible tactile sensor is the piezoresistive effect of the graphene film. When a micro-pressure is applied to the surface of the sensor, the upper bump will collect the stress evenly to the graphene film. This stress will frcture or crack the C–C bond of the graphene film, and the resistivity of the graphene film will change, as shown in Figure 1. Therefore, we can measure the applied micro-pressure according to the varied resistance of the graphene film. Benefiting from the excellent sensitivity and flexibility of graphene film, the sensor can obtain a high sensitivity [17].

### 2.2. Structure

**Figure 2:**
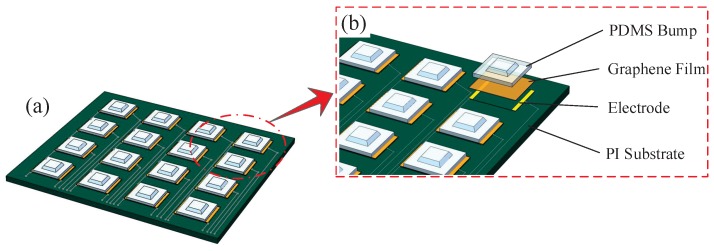
Structure of the electronic skin (**a**); Partial enlarged detail of the electronic skin (**b**).

The structure of the electron skin is shown in Figure 2a. The electron skin is comprised of 4×4 sensing units which contain three layers: The lower substrate, the piezoresistive layer, and the PDMS bump. Figure 2b presents the schematic diagram of the sensor. The lower substrate is composed of electrodes, fabricated by MEMS (micro-electromechanical systems) technology. The piezoresistive layer contains the graphene/polyethylene terephthalate (G/PET) film, which is obtained by the chemical vapor deposition (CVD) method and transferred to the PET substrate by wetting transfer technology. The PDMS bump layer is manufactured by the casting method, and has the ability to collect pressure.

**Figure 3:**
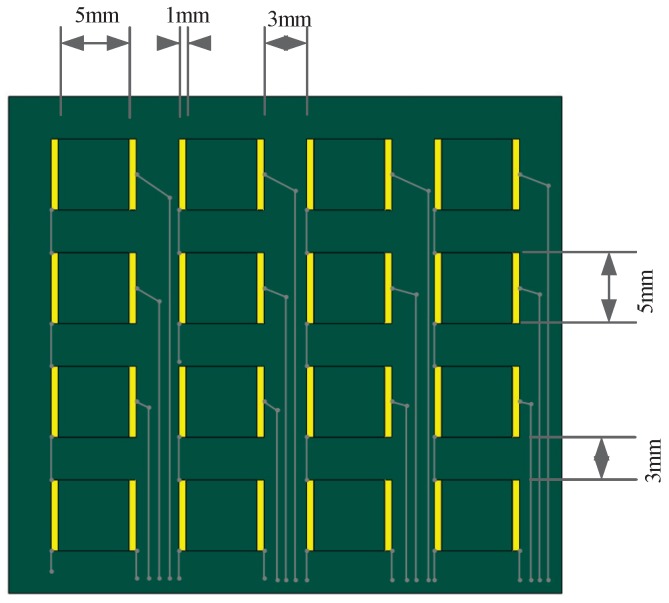
Design of the electronic skin.

The design of the lower substrate is presented in Figure 3. The area of the G/PET films is 5 mm ×5 mm, and the width of the electrodes is 1 mm. Therefore, the area of each sensing unit is 5 mm ×7 mm. This is due to the need for adequate connectivity between the upper and lower substrates. The distance between each sensing unit is 3 mm.

### 2.3. Fabrication Process

**Figure 4:**
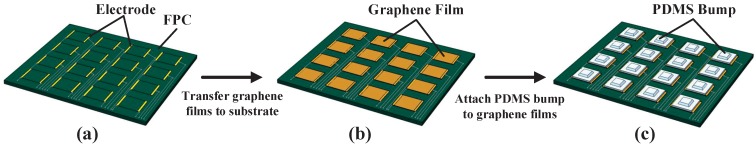
The lower substrate was flexible printed circuit (FPCB) fabricated by MEMS technology (**a**); The graphene films were obtained by CVD method and transfered to the substrate (**b**); The PDMS bump was attched to graphene films (**c**).

**Figure 5:**
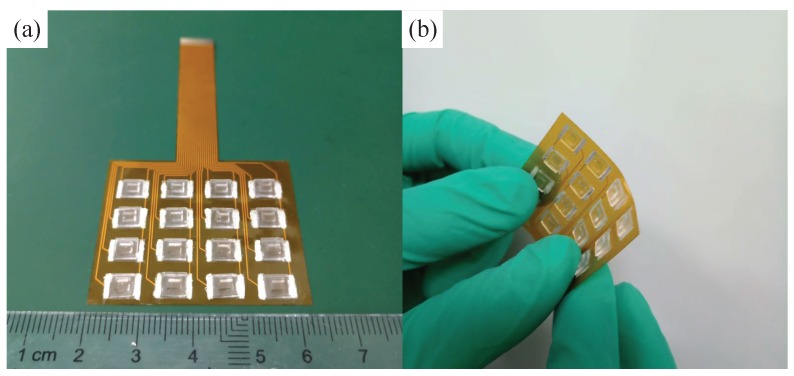
The size of the electonic skins (**a**); Photograph of the electronic skin (**b**).

The fabrication process of the electron skin is presented in Figure 4. First, the lower substrate is flexible printed circuit board (FPCB), which was fabricated by the standard MEMS technology (see Figure 4a): (1) Deposit Cu film by the chemical solution deposition method onto the PI substrate; (2) Cover the photoresistor onto the substrate; (3) Exposure electrode patterns by a photomask; (4) Develop the film and corrode the extra Cu film; (5) Remove the photoresistor. Second, the graphene films were obtained by the CVD method, and transferred to the PET substrate by wetting transfer technology (see Figure 4b). Then, the G/PET was tailored (using scissors) to get the G/PET sensing unit. Third, the sensing unit was attached onto the electrodes through a uniform layer of silver paste, using a brush. The silver paste was solidified in a drying box at $60{\;}^{{}^{\circ}}$C for 2 h. Fourth, the upper PDMS bump was fabricated by the casting method. The PDMS base and curing agent, in the ratio 10:1, were stirred for 10 m. The PDMS solution was poured into the steel mold, and the PDMS was vulcanized for 4 h at $80{\;}^{{}^{\circ}}$C. Fifth, a uniform layer of adhesive PDMS solution was painted onto the middle of the PDMS bump and the G/PDMS films. Then, the adhesive PDMS solution was cured at $80{\;}^{{}^{\circ}}$C for 4 h. In this way, the PDMS bump was attached to the G/PET films, as shown in Figure 4c. The fabricated electronic skin was 43 mm ×37 mm wide and 1.05 mm thick, and the area of the sensing unit was 5 mm ×7 mm, as shown in Figure 5.

### 2.4. Circuit Model

**Figure 6:**
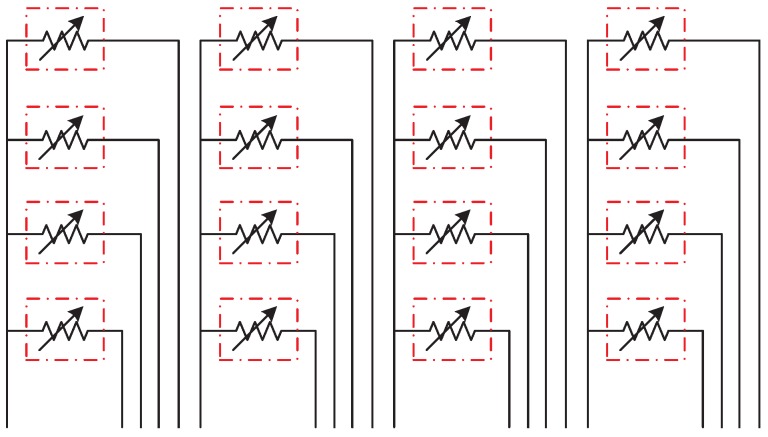
Circuit model of the electronic skin.

Our electronic skin is comprised of 16 sensing units, which can be regarded as rheostats. The resistance of the sensing unit can be changed by applying external pressure. The contact resistance between the G/PET and the electrodes is lower than the resistance of the G/PET. Therefore, the circuit model of the electronic skin can be simplified, as shown in Figure 6.

### 2.5. Measurement Model

When pressure is applied to the surface of the electronic skin, the resistance output of the skin will change, due to the cracks in the G/PDMS films. According to this principle, we can establish a measurement model to measure the pressure. The electronic skin contains 16 sensing units which have two measurement ranges: 0–4 kPa (the small pressure range), and 4–500 kPa (the large pressure range). We use the electronic resistance variation ratios ΔR ($\Delta R=\frac{\left(R_{0}-R_{p}\right)}{R_{0}}$, where $R_{0}$ and $R_{p}$ correspond to the resistance without and with pressure, respectively) as the output of the sensing units. Each of the ranges of the unit are in a positive linear relationship. Therefore, the measurement model of the sensing unit can be defined as

(1) $$\Delta R\left(p\right)=\left\{\begin{array}{cc} ap, & p\in [0,4]\\ cp+d, & p\in [4,500], \end{array}\right.$$

where *a*, *b*, *c*, and *d* are the sensitivities, and the original output is in the ranges of 0–4 kPa and 4–500 kPa, respectively. Therefore, the measurement model of the electronic skin can be given by

(2) $$\Delta R_{ij}\left(p\right)=\left\{\begin{array}{cc} a_{ij}p+b_{ij}, & p\in [0,4]\\ c_{ij}p+d_{ij}, & p\in [4,500] \end{array}\right.\;i,j=(1,2,3,4).$$

In the initial state, the output of the sensing unit is zero; that is, $b_{ij}=0$. Therefore, Equation (2) can be written

(3) $$\Delta R_{ij}\left(p\right)=\left\{\begin{array}{cc} a_{ij}p, & p\in [0,4]\\ c_{ij}p+d_{ij}, & p\in [4,500] \end{array}\right.\;i,j=(1,2,3,4).$$

## 3. Experiment

**Figure 7:**
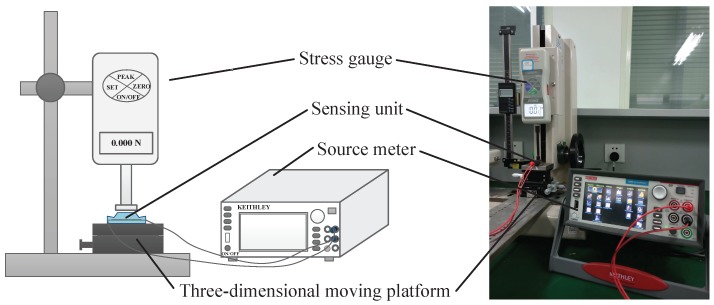
Schematic view and photograph of the experiment.

To carry out the experiment, a home-made pressure measurement platform was constructed, as shown in Figure 7. The platform was comprised of a digital high-precision stress gauge (F1128 ZQ-20A-2, ZHIQU Precision Instruments Co., Hangzhou, China) and a three-dimensional moving platform (LZ 60, Hongjinjie Co., Suzhou, China). The measurement range of the digital high-precision stress gauge is 0–5 N, with an accuracy of 0.001 N. The moving range of the digital high-precision stress gauge in the vertical direction can reach 10 mm with an accuracy of 0.02 mm. The electronic skin was measured by a source meter (Keithley 2450, Tektronix Co., Solon, OH, USA).

## 4. Results and Discussion

### 4.1. Measurement Model

**Figure 8:**
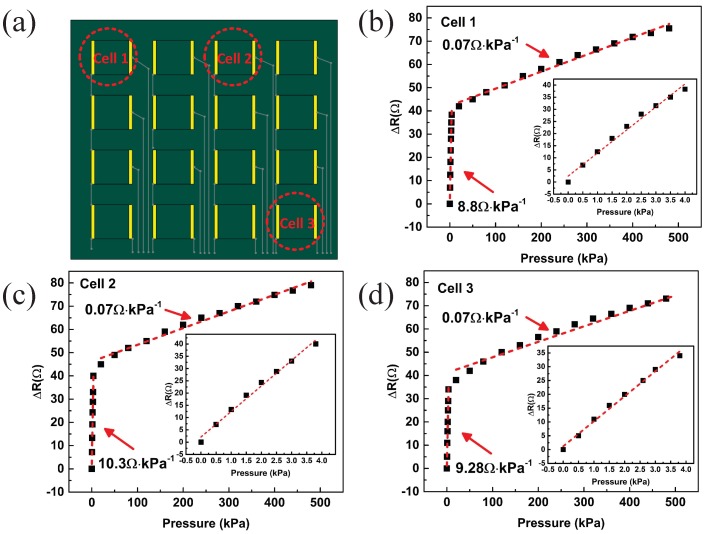
(**a**) Schematic diagram of the location of the sensing unit; (**b**–**d**) Pressure resistance characteristic curves of the sensing units.

According to previous work, we can obtain the measurement model by determining $a_{ij}$, $c_{ij}$, and $d_{ij}$ (i,j=1,2,3,4). Therefore, we measured the output of 3 units ((1,1), (1,3), (4,4)), as shown in Figure 8.

We can obtain $a_{11}$, $c_{11}$, and $d_{11}$ by using the least-squares residual method. With the values obtained, we get

(4) $$\Delta R_{11}\left(p\right)=\left\{\begin{array}{cc} 8.8p, & p\in [0,4]\\ 0.07p+35.2, & p\in [4,500]. \end{array}\right.$$

Similarly, the measurement model of the sensing unit of the positon (1,3) can be written

(5) $$\Delta R_{13}\left(p\right)=\left\{\begin{array}{cc} 9.28p, & p\in [0,4]\\ 0.072p+37.1, & p\in [4,500], \end{array}\right.$$

and the measurement model of the sensing unit of (4,4) can be written

(6) $$\Delta R_{44}\left(p\right)=\left\{\begin{array}{cc} 10.3p, & p\in [0,4]\\ 0.07p+41.2, & p\in [4,500]. \end{array}\right.$$

The measurement model of other sensing units of the electronic skin can be measured by the same method. The electronic skin has the ability to measure not only small pressures (<4 kPa) but also large pressures (>4 kPa).

### 4.2. Sensitivity

Sensitivity is the ratio of the resistance variation ratio (ΔR) to the corresponding variation of the applied pressure (*F*) when the sensor is at a steady state. It is given by

(7) $$S=\frac{\Delta R}{F}.$$

By substituting experimental data into the above equation, we obtain that the sensitivity of the sensing unit of the position (1,1) is 8.8 Ω/kPa in the range of 0–4 kPa, and 0.07 Ω/kPa in the range of 4–500 kPa. Similarly, the sensitivities of the sensing unit of the position (1,3) and (4,4) are 9.28 Ω/kPa and 10.3 Ω/kPa in the range of 0–4 kPa, 0.072 Ω/kPa and 0.07 Ω/kPa in the range of 4–500 kPa.

**Figure 9:**
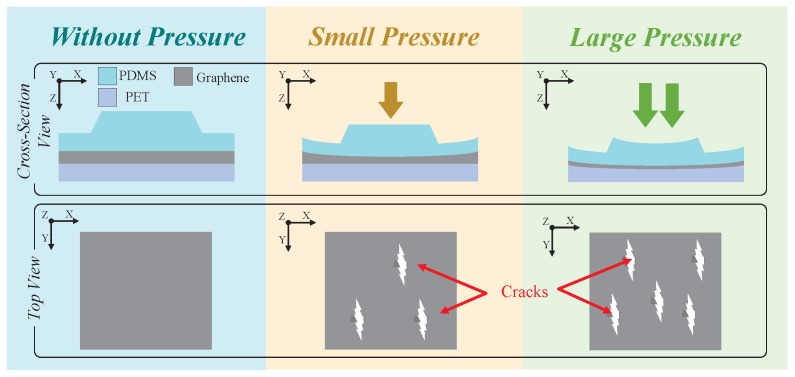
Mechanism of the electronic skin.

The piezoresistive curve showed two segments. The first one is from 0 kPa to 4 kPa, which corresponds to relatively small pressures. A small pressure deforms the PDMS bump, which presses the graphene film and generates many small cracks on the graphene film. Because of the low Young’s modulus of PDMS, a large deformation can be generated by small stress. As a result, the resistance of the sensor in the first segment increased obviously. The second segment is from 4 kPa to 500 kPa, which corresponds to relatively large pressures. In this segment, the deformation of PDMS bump was saturated. However, with the increased pressure, the PET substrate was deformed, generating more small cracks on the graphene film. Due to the high Young’s modulus of PET, a larger pressure is needed to deform the PET, which leads to a reduction in sensitivity. These are shown in Figure 9.

### 4.3. Dynamic Characteristic

**Figure 10:**
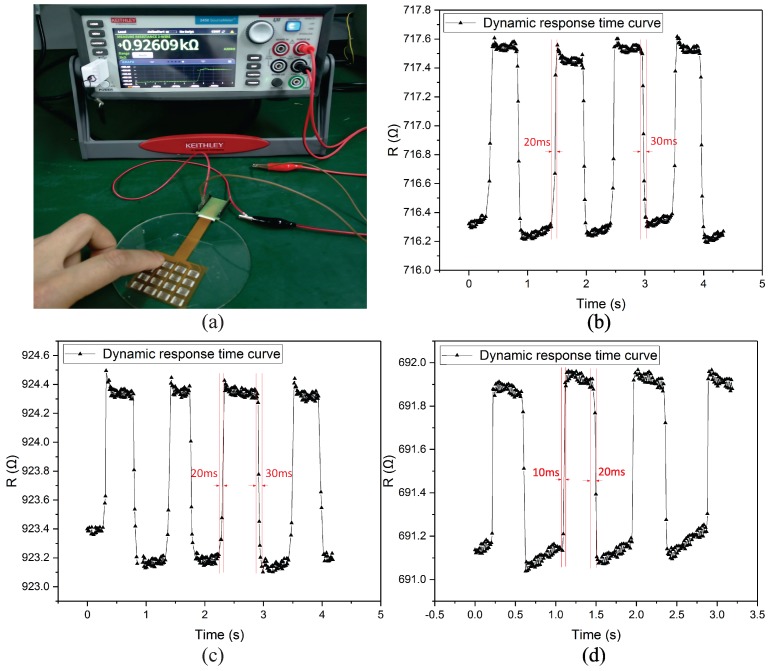
(**a**) Photograph of the measurement of the dynamic characteristic; (**b**–**d**) Measurement results of the sensing units.

As the electronic skin has potential applications in intelligent robot hands and wearable devices, it is important to measure the dynamic characteristics of the electronic skin. We used the source meter to test the dynamic characteristics of the electronic skin. The sampling time of the source meter is 10 ms. The dynamic characteristic was measured with a gentle touch, as shown in Figure 10a.

Figure 10b–d present the dynamic characteristics of the electronic skin. The loading and unloading time of the sensing unit at the position (1,1) were 20 ms and 30 ms, respectively. The measurement results of the sensing unit at the positions (1,3) and (4,4) are shown in Figure 10c,d. We found that the unloading time was larger than the loading time. This is due to the viscoelastic response of the PDMS, which required more time to recover its original shape.

### 4.4. Consistency

**Figure 11:**
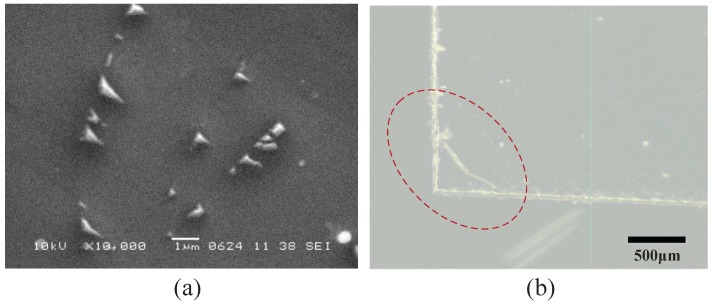
(**a**) SEM micrograph; and (**b**) photograph of the graphene film.

The results in the previous chapter indicated that the outputs of each of the sensing units were not completely consistent. We can find that the inconsistency of the outputs of the sensing units were larger in the range of 0–4 kPa. The differences in the dynamic characteristics are not consistent in the range of 0–4 kPa. To explain this result, the graphene film was investigated at different magnifications, by use of scanning electron microscopy (SEM). We believe that there are two reasons for the inconsistencies: First, the graphene films may be damaged during the transformation process (from Cu film to PET). There are many cracks on the graphene film, as shown in Figure 11a. Second, the graphene films may be ruptured during the tearing process, as shown in Figure 11b. We believe that these reasons lead to the inconsistency of the sensing units.

### 4.5. Application

**Figure 12:**
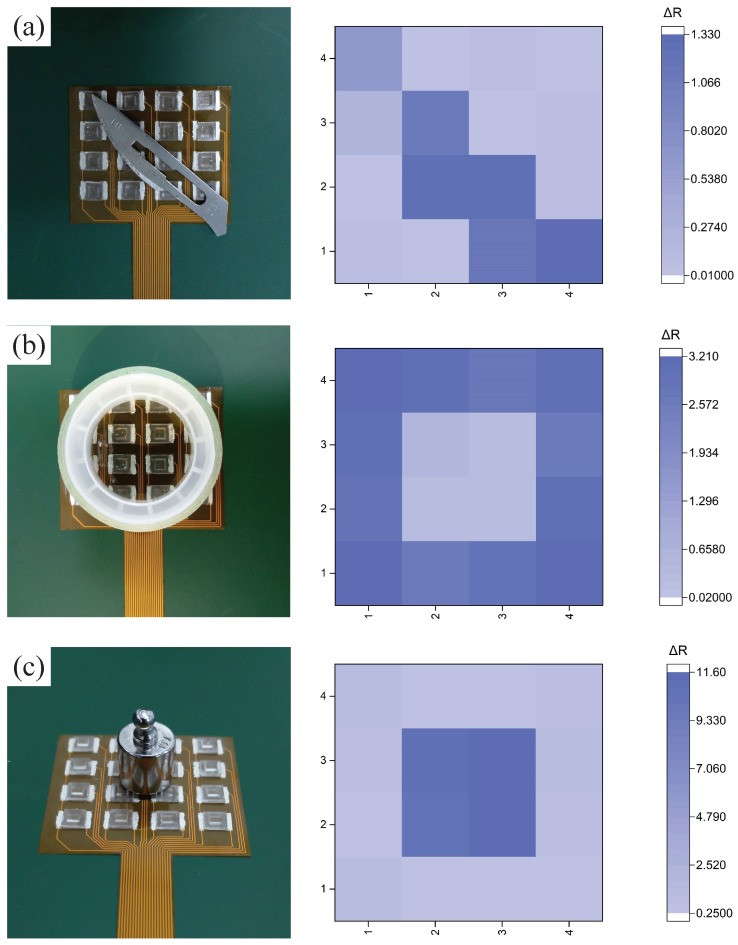
The mapping profile of the pixel signals generated by (**a**) a blade; (**b**) a roll of tape; and (**c**) a weight.

Our electronic skin has the ability to measure not only the pressure, but also the distribution of the pressure. We used a blade (0.885 g), a roll of tape (3.958 g), and a weight (10 g) to test the electronic skin, as shown in Figure 12a–c, respectively.

The results show that the electronic skin could reproduce the appearance of the objects and measure the weight of the objects.

### 4.6. Comparison

Table 1 shows the comparison between the performances of this work and of others. Generally speaking, our electronic skin has high-pressure sensitivity, fast response time, and a large measurement range.

## 5. Conclusions

In this paper, we presented an electronic skin which contains 4×4 sensing units, based on graphene film. Each sensing unit was comprised of PI substrate, graphene film, and a PDMS bump. The dimensions of the sensing unit were 5 mm ×7 mm ×1 mm. The area of the electronic skin was 43 mm ×37 mm. The working mechanism of the electronic skin was analyzed, and the measurement model was established. Using this model, the electronic skin was capable of measuring the normal stress in a range of 0–500 kPa, and of reproducing the appearance of objects. The response time of the electronic skin was 10 ms. By utilizing the piezoresistive effect of the graphene film, our electronic skin had high-pressure sensitivity and a fast response time, and has potential application in the fields of artificial intelligence, robotics, wearable electronics, and prosthetics.

## Figures and Tables

**Table 1:** Comparison between the performances of this work and others.

Reference	Principle	Material	Sensitivity (kPa${}^{-1}$)	Measurement Range (kPa)	Response Times (ms)	Spatial Resolution
This Work	Piezoresistive	G/PET	0.04	0 kPa–500 kPa	20	5 mm
[21]	Transisitor	G/PET	0.12	0 kPa–40 kPa	<10	2.5 mm
[18]	Capacitor	CNT	0.004	0 kPa–50 kPa	125	4 mm
[22]	Piezoresistive	AuNWs	1.14	0 kPa–50 kPa	17	5 mm
[23]	Capacitor	CNT/PDMS	0.198	0 kPa–10 kPa	200	8 mm
